# Supplementary material for: Self-regenerating giant hyaluronan polymer brushes
Source: Nat Commun. 2019 Dec 4;10:5527. doi: 10.1038/s41467-019-13440-7 (PMC6892876; doi:10.1038/s41467-019-13440-7)
Supplement: Supplementary file 1 — Supplementary Information [file 41467_2019_13440_MOESM1_ESM.pdf]

Supplementary Information

Self-Regenerating Giant Hyaluronan Polymer Brushes

W. Wei, Jessica L. Faubel, Hemaa Selvakumar, Daniel T. Kovari, Joanna Tsao, Felipe Rivas, Amar T. Mohabir, Michelle Krecker, Elaheh Rahbar, Adam R. Hall, Michael A. Filler, Jennifer L. Washburn, Paul H. Weigel, Jennifer E. Curtis

t_{growth}	M_n (kDa)	M_w (kDa)	St Dev (kDa)	PDI (M_w/M_n)	Length (μm)	R_H (nm)	# chains
**1 h**	971	1430	665	1.5	3.6	162	2091
**2 h**	1690	3020	1500	1.8	7.5	273	2500
**4 h**	2420	6760	3240	2.8	16.9	450	4823
**8 h**	2410	5130	2560	2.1	12.8	395	3699

Supplementary Table 1. Analysis of the average molecular weight distribution of the HA produced by HA synthase rich membrane fragments. Data was collected using the single molecule nanopore assay at four time points: 1 h, 2 h, 4 h, 8 h. The number and weight averaged molecular weight peaks at 4 hours, with maximal polydispersity (PDI, polydispersity index) at that time point as well. The average length corresponding to the M_w is calculated using 1 nm = 400 Da for the HA polymer. The # of chains is the total number of HA molecules measured for the given brush growth time. The reported standard deviation (st. dev.) captures the width of the distribution. R_H~$M_w^{0.7}$

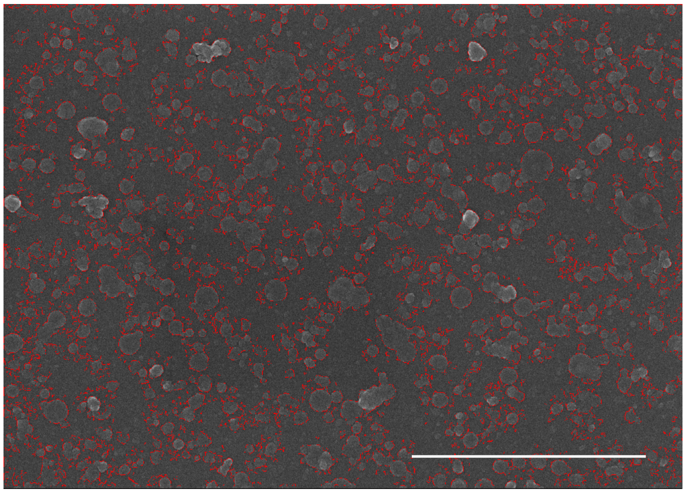

Supplementary Figure 1. Planar glass surface (coverslip) with HA synthase membrane fragments outlined. 2 μm scale bar. Segmentation analysis estimates that ~30% of the surface area is decorated with HA synthase fragments. The average size of the fragments is ~100 nm which is consistent with measurements using dynamic laser scattering.

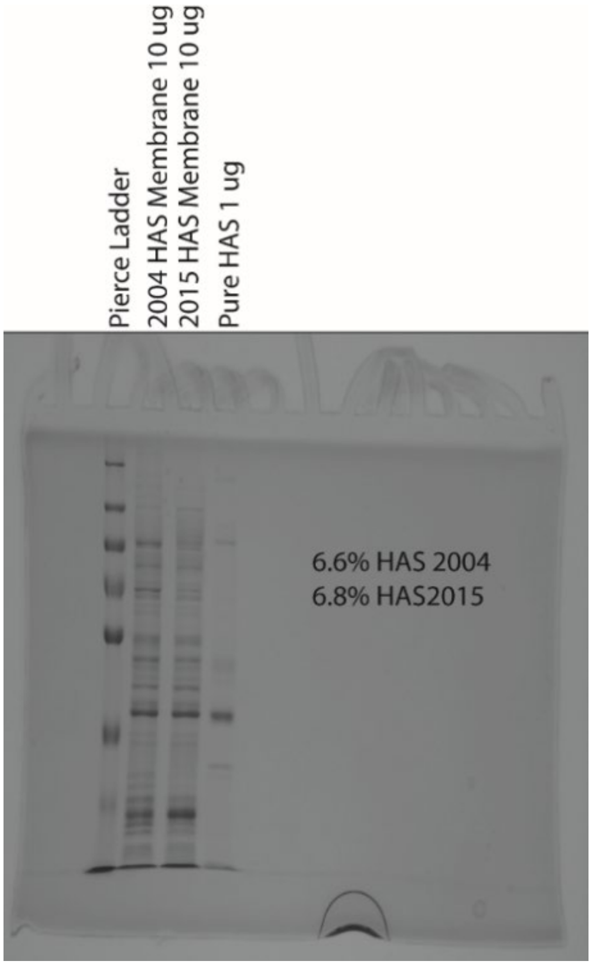

Supplementary Figure 2. Protein content in HA synthase fragments as measured by gel electrophoresis. Gel electrophoresis was performed to determine the total protein content of the HA synthase membrane fragments. Along with membrane fragments that were prepared in 2004 and 2015, purified HA synthase was also run for comparison. By comparing pixel density in each column, it is estimated that 6.8% of the protein present in the 2015 HA synthase membrane fragments are the HAS itself. The 2015 membrane fragment supply was used for all experiments presented in this manuscript [1].

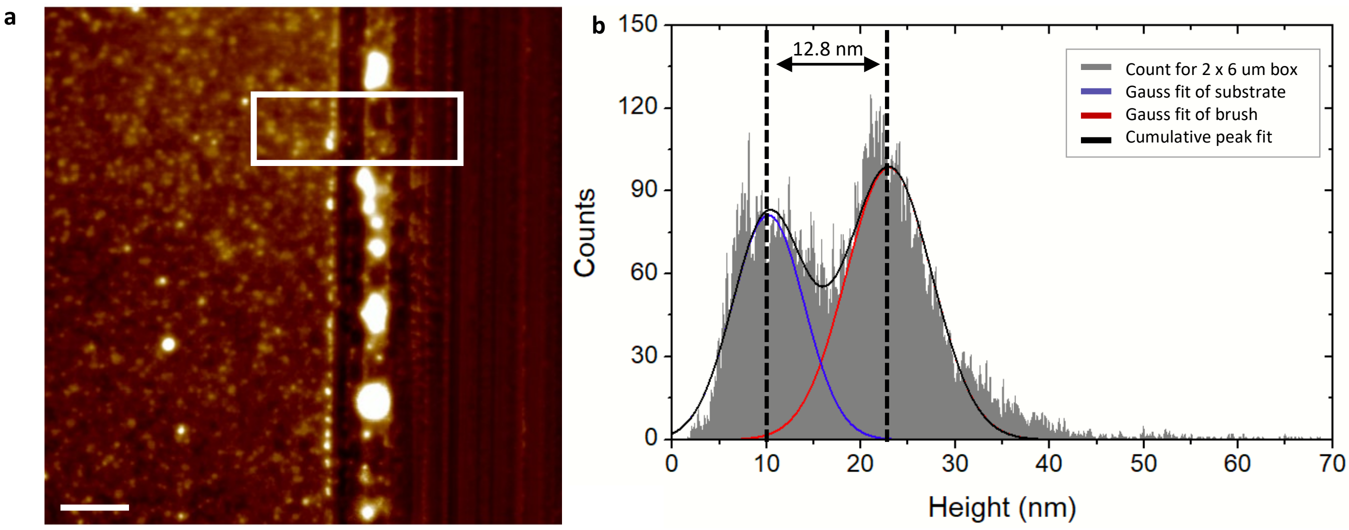

Supplementary Figure 3. Representative AFM result of the brush scratch test. **a,** Topography image with histogram analysis area outlined in white. Scale bar 2 μm. The measurement was repeated on three surfaces in order to acquire an average dry brush height of $H_{dry} = 12.5 \pm 0.7$ nm (st. dev.). **b,** Corresponding height histogram. Z scale = 70 nm.

Supplementary Note 1.
To characterize the shape of the brush concentration profiles, normalized fluorescent (GFPn) profiles in the brush region (defined as from microsphere surface to edge of brush) of spherical brushes were fitted with $\alpha \exp[-r/\beta]$ where r is distance to the surface of the microsphere (in the least square sense). Here, alpha is unitless. Supplementary Fig. 4 shows the typical values for unreinforced brushes on microspheres, while Supplementary Fig. 11 shows the distribution of values for the reinforced brushes on microspheres. In both cases, the brushes were grown for 4 h at 30 °C.

Supplementary Figure 4.

Exponential decay statistics of unreinforced HA brushes on spherical particles (8 μm diameter); $\alpha = 1.09 \pm 0.04$ and $\beta = 1.71 \pm 0.14$ μm where N_{beads}=112, $N_{profiles}$=211. The average R-square value from the fits is 0.988 ± 0.005. All error is st. dev.

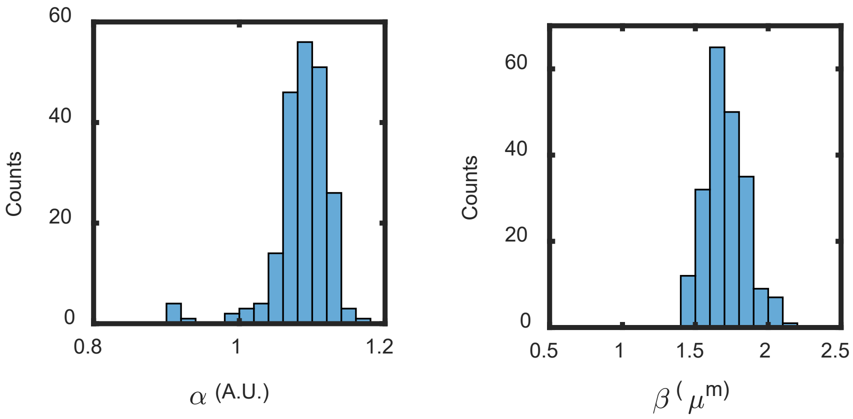

Supplementary Note 2.

We examine the HA brush interface with X-ray photoelectron spectroscopy (XPS) to provide additional evidence that the growing polymer is HA. Importantly, the interface from which the HA brushes grow is complex. It is comprised of glass, poly(ethyleneimine) (PEI), glutaraldehyde (GA), and bacterial membrane fragments, which are rich in membrane proteins. After growth, the HA brush is attached to the surface by HA synthase located in the membrane fragments.

We first investigate surfaces with and without HA to understand the spectral contributions of each component. HA films drop-cast directly on glass exhibit three C(1s) photoelectron peaks near 287.7, 286.0, and 284.3 eV [2, 3], corresponding to O-C-O/O=C-O, C-O, and C-C-/C-H bonds, respectively. A typical C(1s) spectrum acquired in our lab for HA prepared in this manner is shown in Supplementary Figure S5a. Peak values from three HA samples matched those reported in literature, with 287.8, 286.1, and 284.3 eV. The intermediate binding energy peak (C-O) is the most intense at 286 eV.

The glass/PEI/GA/fragment surface from which our HA brush is synthesized, because it is rich in the same chemical bonds as HA, exhibits C(1s) photoelectron peaks with similar binding energies (287.4 eV, 285.8 eV, 284.2 eV) but distinct relative intensities. A typical C(1s) spectrum of a glass/PEI/GA/fragment (i.e., HA-free) surface is shown in Supplementary Fig. S5b. The value of the two highest binding energy peaks (O-C-O/O=C-O and C-O) is small relative to those for HA drop-cast directly on glass. However, the lowest binding energy peak (C-C/C-H) is substantially larger.

A sufficiently thick HA brush on top of the complex glass/PEI/GA/fragment interface would ideally screen the underlying chemistry and produce a spectrum identical to an HA film drop-cast on glass (Supplementary Fig. S5a). However, as can be seen in Fig. S5 d, e, we do not find that this is the case for our HA brushes nor HA dropcast onto the PGF interfaces (Fig. S5c). The underlying glass/PEI/GA/fragment surfaces influence the spectrum.

Change in Area of C-O Peak (286 eV) with HA Addition

	**Change in Area**	**SD**	**N**
**HA Dropcast**	+39,318	1038	3
**4 h Brush**	+14,421	3857	2
**4 h Brush, Reinforced**	+11,778	5036	2

Supplementary Table 2. Change in area under Peak 2 corresponding to C-O (~286 eV) when HA is added to glass/PEI/GA/fragment surfaces either by dropcasting or HA brush growth. The change in area increases with increasing amounts of HA. The PGF average area was obtained from N=4 samples.

To confirm that the polymer brush is HA, we investigate a series of surfaces exposed to HA. Supplementary Figure S5c displays the C(1s) spectrum after drop-casting HA onto the

glass/PEI/GA/fragment surface. As expected, the intensity of the two highest binding energy peaks increases with the addition of HA. However, unlike the case of HA drop-cast on glass (Supplementary Fig. S5a), the lowest binding energy peak from the underlying PEI/GA/fragment surface (arising from C-C/C-H) still dominates the spectrum. We next examine spectra from HA brushes with different known amounts of HA deposited on glass/PEI/GA/fragment surfaces: a 4 h unreinforced brush (Supplementary Fig. S5d) and a 4 h reinforced brush (Supplementary Fig. S5e). Both samples have XPS spectra similar to that of the glass/PEI/GA/fragment/HA dropcast surface (Supplementary Fig. S5c) as opposed to the glass/PEI/GA/fragment surface with no HA (Supplementary Fig S5b). As seen in Table 1, the area of the intermediate binding energy peak (C-O) increases the most for the HA drop-cast directly onto glass. The peak area for C-O also increases for the 4 h brushes, both unreinforced and reinforced. As expected, area increase is less for 4 h brush, than for the drop cast HA. Further, as we might predict, the area increase for the unreinforced brush is larger than for the reinforced 4 h brush. This is consistent with our reports that reinforcement stabilizes the brush but at the cost of some HA loss (see Supplementary Fig. S10).

In summary, XPS data of the HA synthase fragment surfaces with and without HA support the claim that the polymer brushes are comprised of HA. Although the spectrum is not a traditional HA spectrum, due to the underlying complex surface of PEI/GA/fragments, the evolution of the spectrum with addition of HA – specifically the increasing area of peak 2 with increasing HA deposition – confirms a polymer with C-O bonds (e.g. HA) is being deposited on the surface.

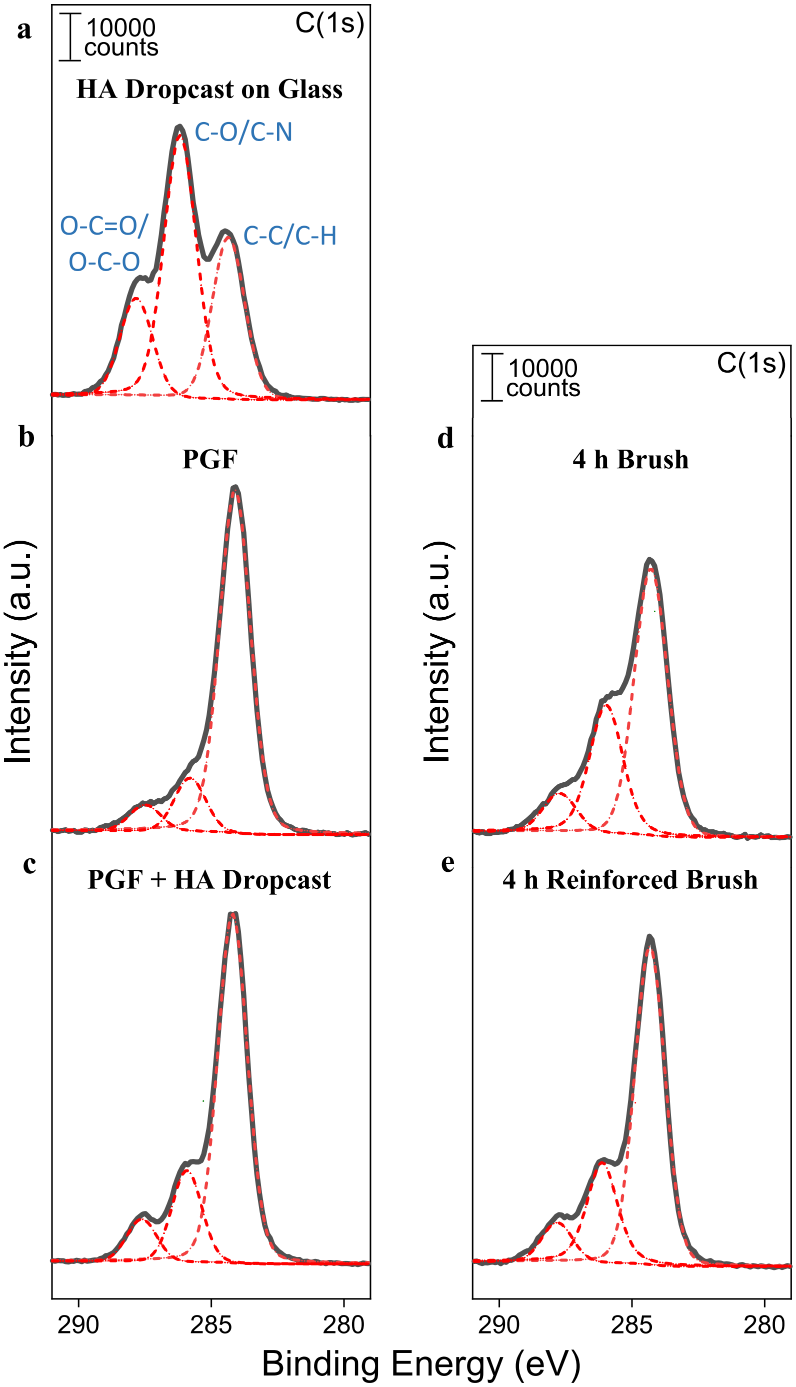

Supplementary Figure 5. Curve fitted C1s XPS spectra obtained for **a,** HA dropcast on glass, **b,** PGF (PEI, GA, membrane fragments only), **c,** HA dropcast on PGF, **d,** 4 h brush, and **e,** 4 h reinforced brush.

Supplementary Note 3.

FluoSpheres (Molecular Probes, Inc, Carboxylate-Modified) were added to a final concentration of 0.007% w/v (green, 20 nm (Catalog number: F8787) and 0.7% w/v red, 200 nm (Catalog number: F8810). Optical characterization of the brush was made using a confocal image on a scanning laser confocal microscope (FV1000, Olympus, Tokyo, Japan; Objective: PlanApo N, 60X/1.42 NA oil) for high-resolution confocal images. When imaging planar polymer brushes, we use a 100 nm vertical step. A 20 µm thick z-stack is taken of the planar brush. The intensity profiles of the green and red beads are plotted (Figure S4). The green profile corresponding to the smaller beads (20 nm) peaks at the surface of the sample. The red profile requires a linear fit of the intensity decay at high z positions above the object to correct for aberrations and absorption. The red profile is then corrected using this linear decay by normalization. The z-location of the 50% intensity value of the red profile is taken to be the average edge of the brush. Finally, the difference in the number of z-slices from the z-stack of the peak location of the green profile (the surface) and the 50% intensity value in the red profile (the average edge of the brush) is the thickness. Then, given that the z-stack is taken in 100 nm thick steps, one can convert the number of slices to a brush thickness value in microns.

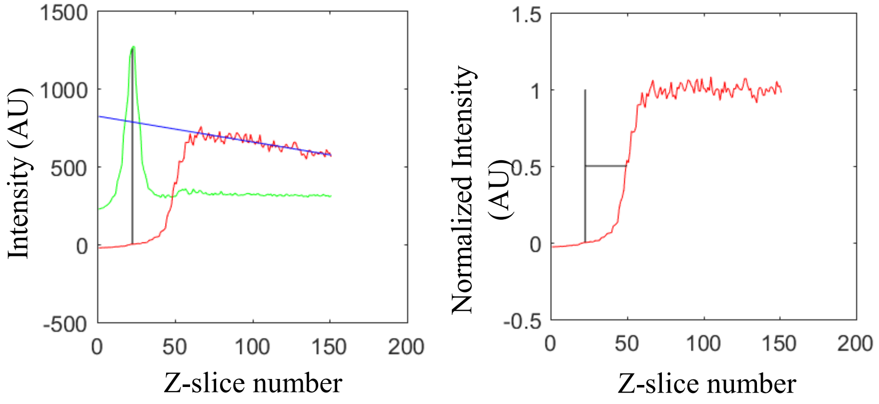

Supplementary Figure 6. **(Left)** Intensity profiles of green (20 nm) and red (200 nm) beads interacting with planar HA brush. The black vertical line denotes the surface and the blue line is the linear fit of the intensity decay in the red beads. **(Right)** The normalized red bead intensity is plotted along with the same black vertical line denoting the surface. The difference in the number of slices in the z-stack between the black vertical line and the 50% intensity value in the red bead plot is used to determine the thickness of the brush.

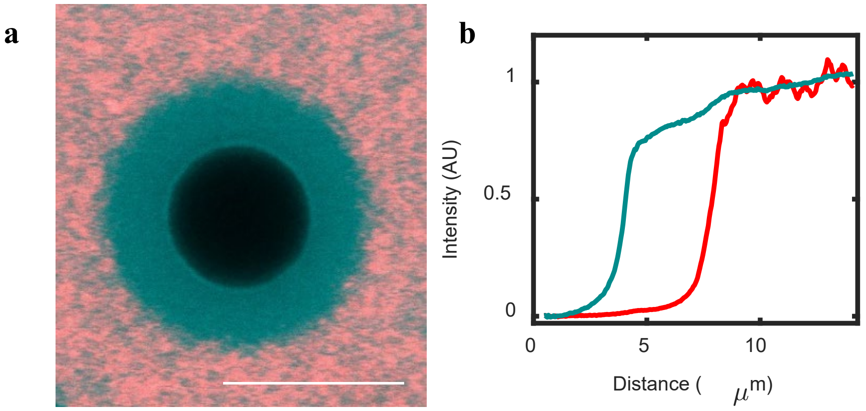

Supplementary Figure 7. a, 100 nm particle penetration particle exclusion assays (scale bar 10 μm) and **b**, profiles of particle penetration (red) and dextran (cyan, 10 kDa). The penetration of 100 nm is less sharp than the 200 nm particles (shown in Fig. 2d of the manuscript [1]); however, they still remain roughly at the edge of the brush. The thickness of the spherical brush as measured with 100 nm particles is 3.4 ± 0.2 μm (N=52, st. dev.), whereas the thickness when measured with 200 nm particles is 3.8 ± 0.3 μm (N=148, st. dev.).

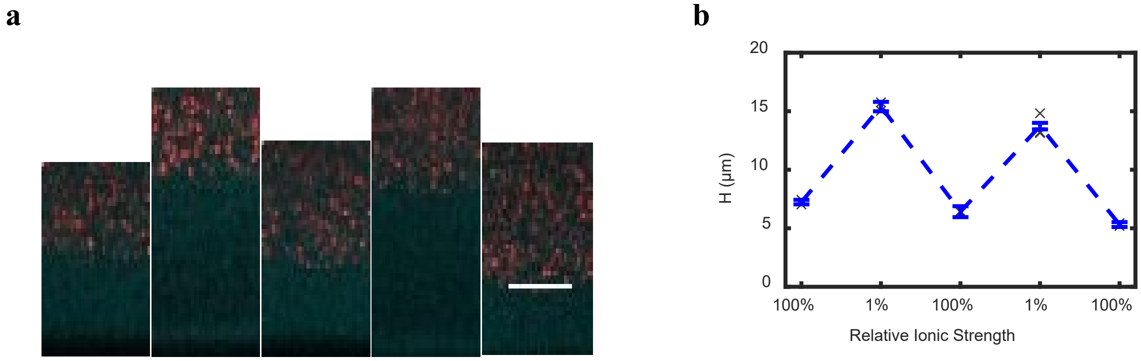

Supplementary Figure 8. **a**, XZ profile of a HA brush's stimulus responsiveness and reversibility to ionic strength swapping; switching from 100% to 1% dilution of the imaging buffer with deionized water. (The ionic strength was diluted from ~130 mM to ~1.3 mM). In contrast to the extreme example displayed in Figure 3 in the paper, this shorter brush (t_{growth}=4 h rather than t_{growth}=15 h) stretches only to ~15 μm (from ~7 μm) rather than 22 μm; but it also is more reversible, losing less height with each solvent swap (scale bar 10 μm). **b**, Height measurements from (a) for 5 regions of the sample reported by grey x's (region area was 211 x 211 μm^2). The blue shows mean and st. dev of the measurements on the same brush.

Supplementary Note 4

We tested the impact on brush height of exchanging solvents. Since the HA is not permanently bound to the HA synthase, some HA loss is expected. Every 20 min for 1 h, while the sample sat on the confocal microscope stage at 30 °C, six gentle pipette pumps were administered to the sample (18 total). The effect was to mimic conditions of exchanging solvents. Over the course of one hour and 18 gentle pipette pump actions, the average height of the planar brushes was relatively stable (Figure S10), showing a slight decrease in average values. This induced height decrease is more significant than for a brush measured every 20 min with no solvent swaps (see Figure 4d in manuscript [1]). Hence shear induced desorption driven by pipetting increases brush decay relative to the decay from natural desorption.

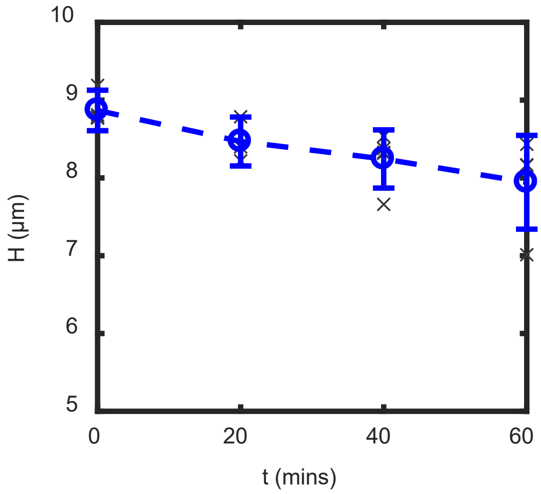

Supplementary Figure 9. Each blue circle represents the average brush height after 6 gentle pipetting pumps (t_{growth}=5.5 h) for one brush. Error bars report st. dev. The grey x's are the height measurements on the same brush from at least four unique areas on the same sample.

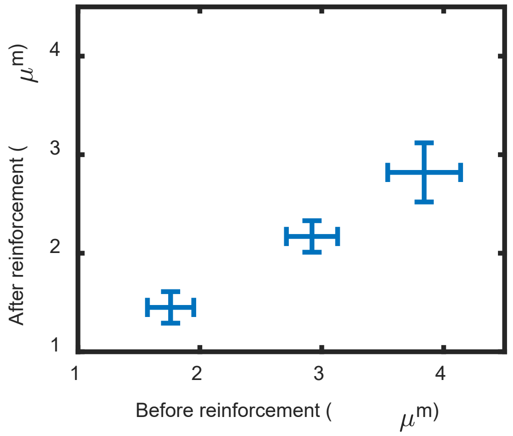

Supplementary Figure 10. Brush thickness before and after reinforcement. Reinforcing the brush by covalent binding to the interface reduces the brush height by ~34% on average. The slope of a line fit through the data is 0.66. Error bars show st. dev. N > 120 for unreinforced brushes at each time point (t = 1, 2, 4 h); for the reinforced brushes, the number of particles measured at each time were N = 72, 42, 35.

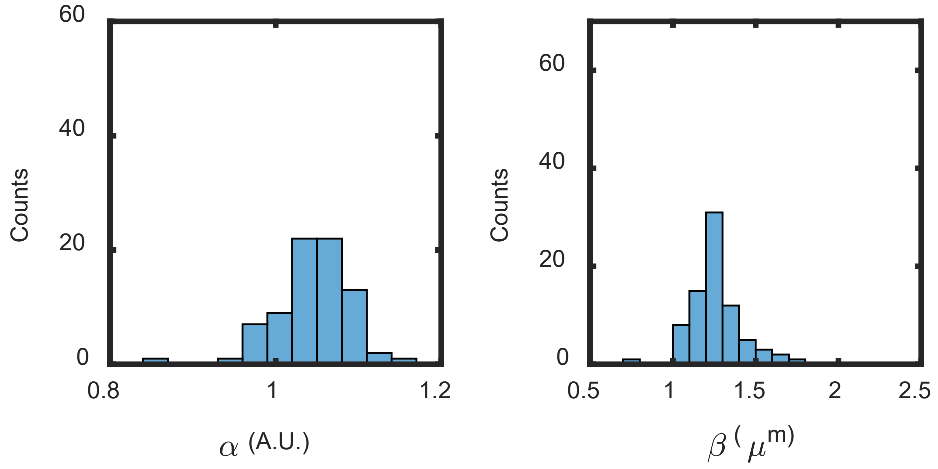

Supplementary Figure 11. Exponential decay statistics of reinforced HA brushes on spherical particles (8 μm diameter) measured by fluorescent labeling with GFPn; $\alpha = 1.04 \pm 0.05$ (st. dev.) and $\beta = 1.26 \pm 0.15$ μm (st. dev.) where N_{beads}=81, $N_{profiles}$=360. The average R-square value from the fits is 0.992 ± 0.009.

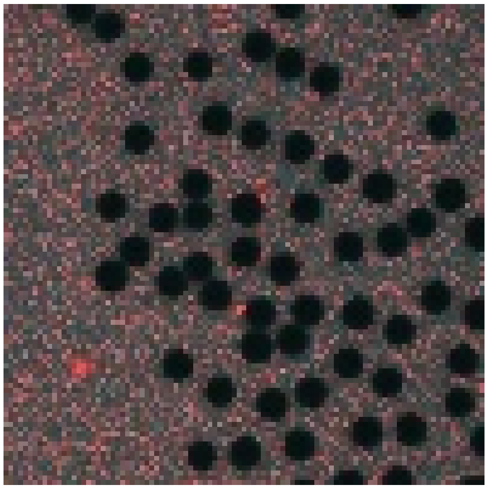

Supplementary Figure 12. Reinforced brushes on spherical particles are digested by hyaluronidase, as shown by the red 200 nm particles reaching the surface of the larger 8 μm spheres after enzymatic removal of the brush.

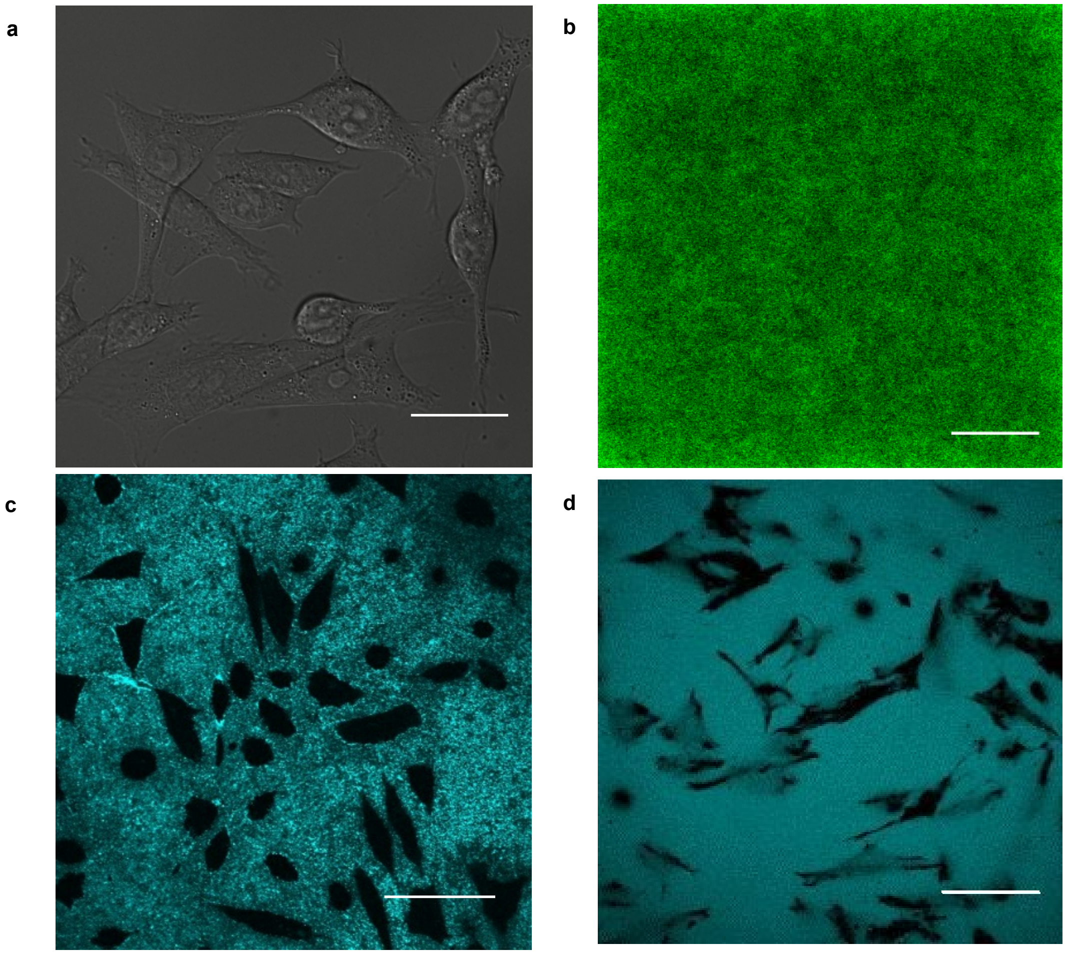

Supplementary Figure 13. a, Fibroblasts locally digest the HA brush and adhere to the underlying glass interface. Scale bar 25 µm. **b,** Fluorescently-labeled brush before fibroblasts digest and adhere to underlying surface (no tracks visible; compare with Figure 6a in main manuscript). Scale bar 10 µm. c, Fluorescent dextran (cyan) is excluded from area underneath adherent cells on surfaces with no HA brush (cells are on glass/PEI/GA/bacteria fragment surface with no brush). Scale bar 50 µm. **d**, Dextran highlights space under adherent cells on reinforced HA brush (t_{growth}= 4 hr). Black regions correspond to cell area in direct contact with surface. Scale bar 50 µm.

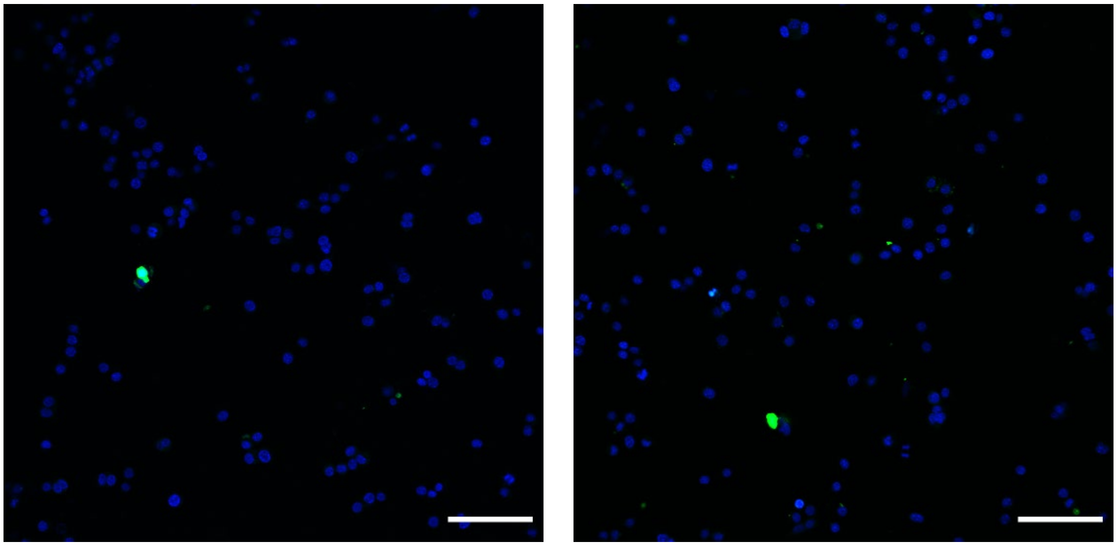

Supplementary Figure 14. Cell viability assay. Two representative laser confocal images showing fluorescence from live and dead cell assay kit. Blue demarcates all cells, while green labels only dead cells. A total of 6 dead cells were identified among ~3000 identified cells. Scale bars are 100 μm.

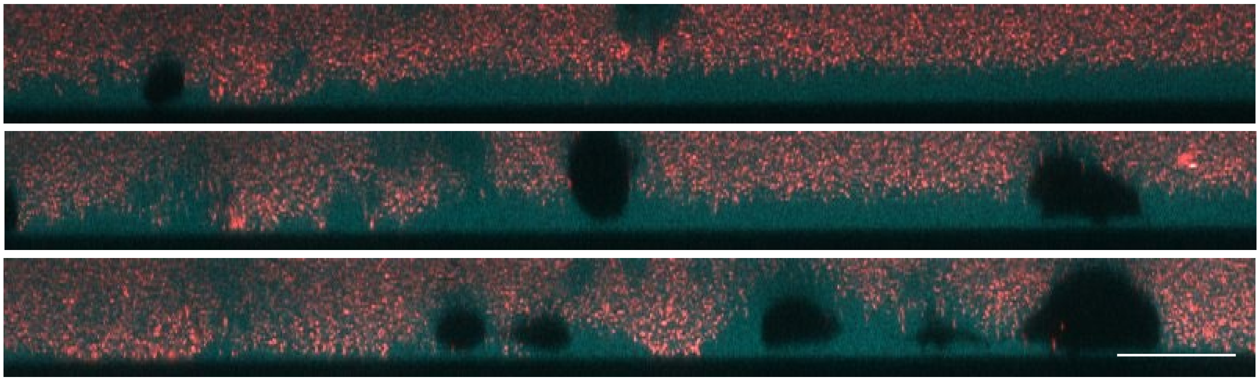

Supplementary Figure 15. Three side view confocal images taken from the sample shown in Figure 6b in reference [1] (the corresponding manuscript). Regions where red nanoparticles touch the glass substrate (the black at the bottom of the images) lack brush. Cell cross-sections are the black shapes above the glass substrate. These particle exclusion assays of the cell-brush interface at 12 h confirm that portions of the brush have been removed during the cell interactions, presumably by cell-mediated hyaluronidase activity. Scale bar 20 μm.

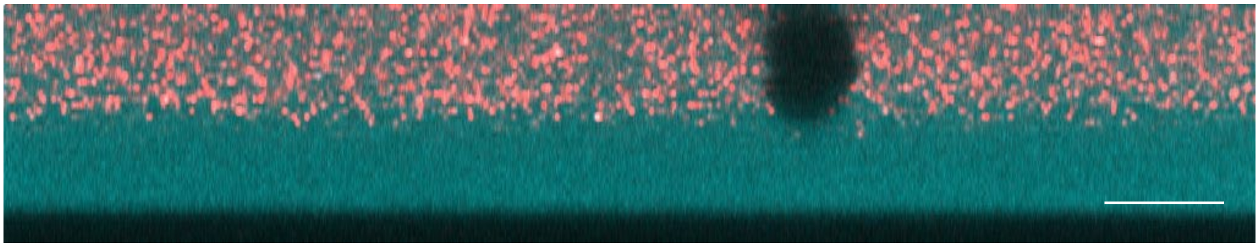

Supplementary Figure 16. Eight micron glass microspheres (ρ_{silica}=2.65 g/cm^3> ρ_{cell}~1.1 g/cm^3 [4]) remain suspended above a 4 h brush demonstrating that cell access to underlying substrate requires active mechanism such as digestion of HA brush or cellular adhesion and forces to compress the brush, or both. 10 µm scale bar.

Retainment of Bacteria in Biofilms on Different Substrates

	# of bacteria	Glass	Film	Brush
1 day	Mean	4518	271	43
	SEM	567	33	3
5 days	Mean	752	224	32
	SEM	58	33	3

Supplementary Table 3: Total number of PAO1 bacteria post-wash on glass, HA film and HA brush in a 211 x 211 x 5 um^3 volume averaged over 5 measured areas.

% Retainment of Bacteria in Biofilms on Different Substrates

	% (rel. glass)	Glass	Film	Brush
1 day	Mean	100%	6.0%	0.95%
	SEM	13.0%	0.7%	0.07%
5 days	Mean	100%	30%	4.3%
	SEM	7.8%	4.4%	0.34%

Supplementary Table 4: Relative percentage PAO1 bacteria adherent to HA film and HA brush post-washing as compared to the bacteria sticking to the glass substrate.

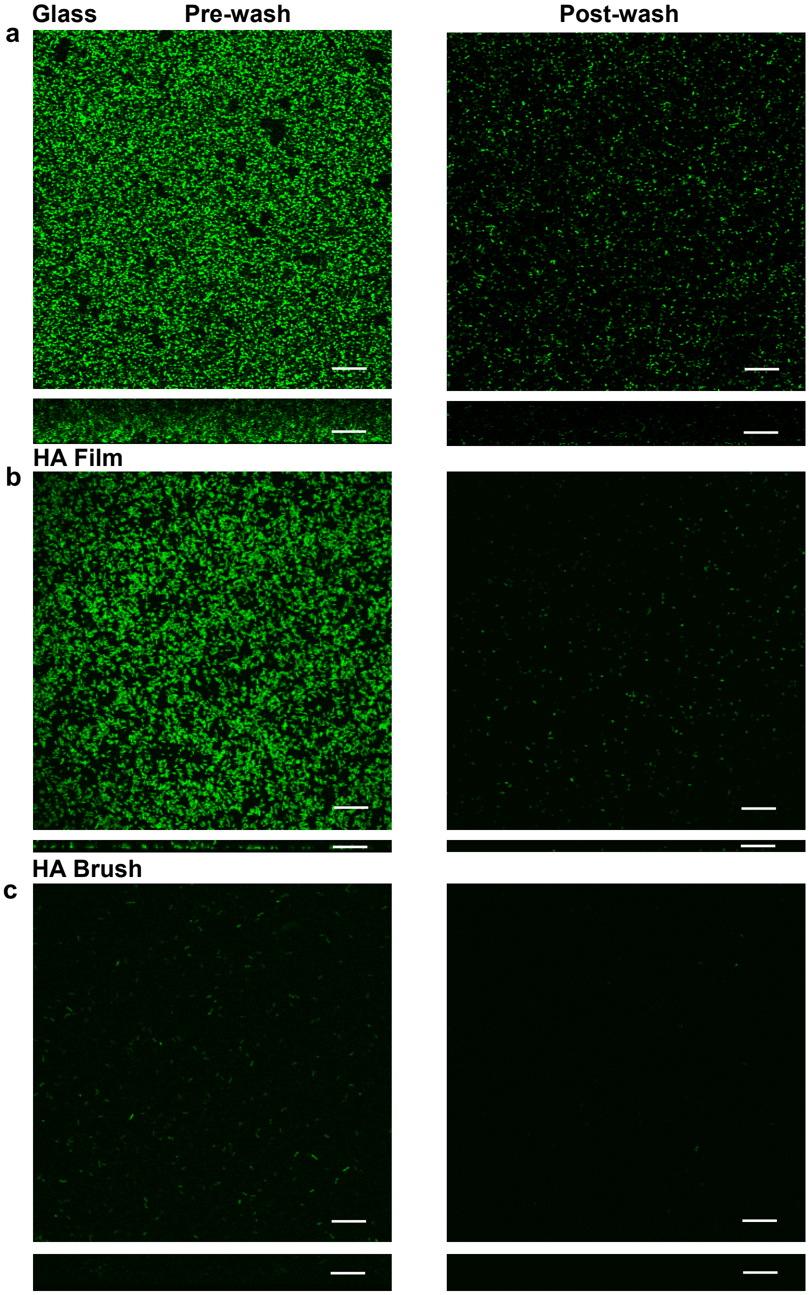

Supplementary Figure 17. **a, b, c,** Wide-field of view confocal micrographs of GFP-producing *Pseudomonas aeruginosa* (PAO1) interacting with glass (**a**), HA film (**b**), and reinforced HA brush (**c**). Left: biofilm growth before washing (1 day). Right: biofilm after washing (1 day). XZ side views of the biofilms are presented below each respective XY top view of the samples. Scale bars, 10 µm. Dextran was used to identify the glass interface beneath the brush (not shown above).

References

[1] Wei, Wenbin, *et al.* “Self-Regenerating Giant Hyaluronan Polymer Brushes.” *Nature Commun*. (in press).

[2] Kitching, K. J., et al. "Development of an electrospray approach to deposit complex molecules on plasma modified surfaces." *Review of scientific instruments* 74.11 (2003): 4832-4839.

[3] D’Sa, Raechelle A., et al. "Protein, cell and bacterial response to atmospheric pressure plasma grafted hyaluronic acid on poly (methylmethacrylate)." *Journal of Materials Science: Materials in Medicine* 26.11 (2015): 260.

[3] Grover, William H., *et al.* "Measuring single-cell density." *Proceedings of the National Academy of Sciences* 108.27 (2011): 10992-10996.
