# Supplementary material for: Self-regenerating giant hyaluronan polymer brushes
Source: Nat Commun. 2019 Dec 4;10:5527. doi: 10.1038/s41467-019-13440-7 (PMC6892876; doi:10.1038/s41467-019-13440-7)
Supplement: Supplementary file 3 — Description of Additional Supplementary Files [file 41467_2019_13440_MOESM3_ESM.pdf]

Description of Additional Supplementary Files

File Name: Supplementary Movie 1
Description: MEF interaction with HA brush.avi Mouse embryonic fibroblasts (MEFs) interacting with HA brush over 12 h. In the beginning the cells are out of focus, hovering above the brush. After several hours, the cells digest the brush and adhere to the underlying substrate where they begin to proliferate.
