# Supplementary material for: Self-regenerating giant hyaluronan polymer brushes
Source: Nat Commun. 2019 Dec 4;10:5527. doi: 10.1038/s41467-019-13440-7 (PMC6892876; doi:10.1038/s41467-019-13440-7)
Supplement: Supplementary file 5 — Reporting Summary [file 41467_2019_13440_MOESM5_ESM.pdf]

nature research

Corresponding author(s): Jennifer Curtis

Last updated by author(s): 19-11-04

Reporting Summary

Nature Research wishes to improve the reproducibility of the work that we publish. This form provides structure for consistency and transparency in reporting. For further information on Nature Research policies, see Authors & Referees and the Editorial Policy Checklist .

Statistics

For all statistical analyses, confirm that the following items are present in the figure legend, table legend, main text, or Methods section.

n/a	Confirmed	
☐	☒	The exact sample size (n) for each experimental group/condition, given as a discrete number and unit of measurement
☐	☒	A statement on whether measurements were taken from distinct samples or whether the same sample was measured repeatedly
☒	☐	The statistical test(s) used AND whether they are one- or two-sided<br>*Only common tests should be described solely by name; describe more complex techniques in the Methods section.*
☒	☐	A description of all covariates tested
☒	☐	A description of any assumptions or corrections, such as tests of normality and adjustment for multiple comparisons
☐	☒	A full description of the statistical parameters including central tendency (e.g. means) or other basic estimates (e.g. regression coefficient) AND variation (e.g. standard deviation) or associated estimates of uncertainty (e.g. confidence intervals)
☒	☐	For null hypothesis testing, the test statistic (e.g. F, t, r) with confidence intervals, effect sizes, degrees of freedom and P value noted<br>*Give P values as exact values whenever suitable.*
☒	☐	For Bayesian analysis, information on the choice of priors and Markov chain Monte Carlo settings
☒	☐	For hierarchical and complex designs, identification of the appropriate level for tests and full reporting of outcomes
☒	☐	Estimates of effect sizes (e.g. Cohen's d, Pearson's r), indicating how they were calculated

Our web collection on statistics for biologists contains articles on many of the points above.

Software and code

Policy information about availability of computer code

Data collection

Software for Olympus Confocal Microscope: FV10-ASW Version 04.02.03.06

Detailed protocol for data collection/analysis for HA molecular weight distribution are given in the published manuscript by co-author Hall et al, 'Label-free analysis of physiological hyaluronan size distribution with a solid-state nanopore sensor', Nature communications 9 (1), 1037.

AFM data collection:
Images taken with Research Nanoscope 8.15 (Build R3.87387).
Image analysis and height histograms obtained with Bruker NanoScope Analysis 1.4 (Build R2Sr1.83411).
Peak fitting with OriginPro 8.5.0 SR1 b161 (Academic)

XPS:
XPSPeak 41 for fitting spectra
Plotting Origin pro 2019b(9.65)

Data analysis

All programs described below that were used to analyze data are written using MATLAB R2016b. Much more detail regarding analysis is presented in the Methods Section and the Supplementary Data.

The codes are available at https://github.com/wwb203/HyaluronanNatureCommunication
The core portion of the code are described below and provided at the link. In addition, the code that is used to produce output is in another folder called 'codeUsedForResearch'. That folder has test images (.oib files), a ReadMe file and the full code itself.

1. Spherical Brush Thickness Analysis (sphericalBrushParticleExclusionAssay.m): The code identifies the xy slice with the largest glass bead

radius (from a z-stack), then generates the azimuthally-averaged intensity profile, and locates the brush surface and the brush edge. This program is used to analyze particle distributions along the radial direction as well as to find fluorescence profile of GFPn labeled brushes. The output depends on the input (brush height; nanoparticle profile, fluorescence profile).

2. Planar Brush Thickness Analysis (planarBrushParticleExclusionAssay.m): The code analyzes a z-stack of a brush to find the average intensity profile in the z direction of whatever object is present (nanoparticles, dextran, GFPn). It also identifies the glass substrate and the edge of the excluded 200nm beads and uses the calibrated distance to estimate the height of the brush.

3. Brush Fluorescence Analysis (GFPnProfileAnalysis.m): Analyzes a fluorescent profile taken either from a spherical or planar brush and extracts the thickness of the brush as determined by the fluorescent profile.

For manuscripts utilizing custom algorithms or software that are central to the research but not yet described in published literature, software must be made available to editors/reviewers. We strongly encourage code deposition in a community repository (e.g. GitHub). See the Nature Research guidelines for submitting code & software for further information.

Data

Policy information about availability of data

All manuscripts must include a data availability statement. This statement should provide the following information, where applicable:

- Accession codes, unique identifiers, or web links for publicly available datasets
- A list of figures that have associated raw data
- A description of any restrictions on data availability

Data for all figures in the manuscript and the supplemental materials are provided online at https://github.com/wwb203/HyaluronanNatureCommunication

Field-specific reporting

Please select the one below that is the best fit for your research. If you are not sure, read the appropriate sections before making your selection.

- [x] Life sciences
- [ ] Behavioural & social sciences
- [ ] Ecological, evolutionary & environmental sciences

For a reference copy of the document with all sections, see nature.com/documents/nr-reporting-summary-flat.pdf

Life sciences study design

All studies must disclose on these points even when the disclosure is negative.

Sample size	Sample sizes were determined by the number of data points collected per measurement - the number of planar surfaces measured, the number of locations per planar surface that was measured, the number of microbeads analyzed, the number of biofilms studied, the number of locations per biofilm analyzed, the number of events.
Data exclusions	We know brush height is highly reproducible from making 100s of measurements for various controls, experiments, training numerous undergraduate students, etc. Occasionally a sample produces a brush that is out of range of the standard deviation (~1 in 20 samples). In the data collection for this manuscript, this occurred once and rather than continue with the sample, we chose to use another sample from the same sample preparation. In all cases however, we emphasize that we provide triplicates and there was only this one situation where data was excluded.
Replication	All data points were collected in triplicate (at minimum) for the crucial results. The three exceptions where only one sample was measured in multiple places are: 1) ionic strength swapping; 2) brush growth pausing and restarting and 3) desorption of HA from unreinforced brushes. The protein content gel for the Sure 2 cell line was only performed once, as is standard.
Randomization	N/A. Randomization was not relevant to our study because all coverslips come from the same source and are considered to be identical. There are no experimental groups differentiating the bacteria that produced the HAS fragments, the P. aeruginosa used for biofilm formation, or the MEF cells.
Blinding	Blinding was not relevant to our study.

Reporting for specific materials, systems and methods

We require information from authors about some types of materials, experimental systems and methods used in many studies. Here, indicate whether each material, system or method listed is relevant to your study. If you are not sure if a list item applies to your research, read the appropriate section before selecting a response.

Materials & experimental systems

n/a	Involved in the study	
x		Antibodies
	x	Eukaryotic cell lines
x		Palaeontology
x		Animals and other organisms
x		Human research participants
x		Clinical data

Methods

n/a	Involved in the study	
x		ChIP-seq
x		Flow cytometry
x		MRI-based neuroimaging

Eukaryotic cell lines

Policy information about cell lines

Cell line source(s)	Vinculin-null mouse embryonic fibroblasts (MEFs) were from Eileen Adamson (Burnham Institute, La Jolla, CA). Subsequently, vinculin null MEFs were transduced with retrovirus to express the pXF40-eGFP-vinculin vector, as described previously in DW Dumbauld, et al, How Vinculin Regulates Force Transmission, PNAS 110 (24), 9788-9793, https://www.pnas.org/content/110/24/9788.
Authentication	Expression of eGFP-WT-Vinculin was validated using western blotting, confocal imaging, and fluorescence activated cell sorting (FACS).
Mycoplasma contamination	The cells were tested for mycoplasma and they are free of mycoplasma contamination.
Commonly misidentified lines (See ICLAC register)	None of the cells is listed in ICLAC.
